# The Controversy Persists: Is There a Qualification Criterion to Utilize Inhaled Nitric Oxide in Pre-term Newborns?

**DOI:** 10.3389/fped.2021.631765

**Published:** 2021-03-31

**Authors:** Frederico Vieira, Marjorie Makoni, Edgardo Szyld, Krishnamurthy Sekar

**Affiliations:** Neonatal Perinatal Section, Department of Pediatrics, University of Oklahoma Health Sciences Center, Oklahoma City, OK, United States

**Keywords:** pre-term infants, pulmonary hypertension, cardiovascular, inhaled nitric oxide, inhaled nitric oxide (iNO), persistent pulmonary hypertension of the newborn

## Abstract

Inhaled nitric oxide (iNO) use in premature newborns remains controversial among clinicians. In 2014, the American Academy of Pediatrics, Committee on Fetus and Newborn released a statement that the available data do not support routine iNO use in pre-term newborns. Despite the absence of significant benefits, 2016 California data showed that clinicians continue to utilize iNO in pre-term infants. With studies as recent as January 2017, the Cochrane review confirmed no major advantages of iNO in pre-term newborns. Still, it recognized that a subset of pre-term infants with pulmonary hypertension (PHTN) had not been separately investigated. Furthermore, recent non-randomized controlled trials have suggested that iNO may benefit specific subgroups of pre-term newborns, especially those with PHTN, prolonged rupture of membranes, and antenatal steroid exposure. Those pre-term infants who showed a clinical response to iNO had increased survival without disability. These findings underscore the need for future studies in pre-term newborns with hypoxemic respiratory failure and PHTN. This review will discuss the rationale for using iNO, controversies regarding the diagnosis of PHTN, and additional novel approaches of iNO treatment in perinatal asphyxia and neonatal resuscitation in the pre-term population < 34 weeks gestation.

## Impact Statement

- The report aims to explain why neonatologists prescribe inhaled nitric oxide to premature newborns, despite recommendations against it.
- The article reviews biological factors that may predispose premature newborns to benefit from inhaled nitric oxide.
- The manuscript takes a different approach from systematic reviews. It discusses non-randomized controlled trials to analyze the rare events of pulmonary hypertension in pre-term newborns that appropriately responded to inhaled nitric oxide.

## Introduction

Nitic oxide (NO) is a known free radical important as a chemical intermediate in multiple environmental reactions (1–4). In the 1990's, Nitric oxide's function was better elucidated as an endothelium-derived relaxing factor and signaling molecule (5, 6). Later, many investigations targeted NO in the cardiovascular, nervous, immune, and gastrointestinal systems, demonstrating its significant regulatory effects in physiological and pathophysiological processes (7–14).

Nitric oxide has been extensively investigated in the fetus and newborn due to its ability to influence basal pulmonary vascular tone (15–17). Term and late-pre-term newborns with poor postnatal vascular adaptation, leading to pulmonary hypertension of the newborn and hypoxemic respiratory failure (HRF), experienced significant benefits after inhaled nitric oxide (iNO) treatment. Systematic reviews confirmed a decreased need for extracorporeal membrane oxygenation (ECMO) and related complications after iNO (18). In 1999, the US Food and Drug Administration approved iNO therapy for HRF in term and late pre-term newborns (19). Subsequently, researchers investigated the benefits of iNO in pre-term infants. The investigations focused on iNO use in reducing chronic lung disease development in extremely premature infants. These randomized controlled trials showed mixed results, and no consensus has been achieved for recommending iNO use in pre-term infants <34 weeks gestation. This review discusses the findings and controversies regarding iNO administration in pre-term newborns. A focused PubMed^®^ search with the term *nitric oxide* appearing with either *pre-term* or *premature* resulted in 272 articles through February 2020. Case report and case series were excluded. Retrospective case controls, comparative or larger population-based studies were the primary source of this analysis showing clinical improvement after iNO exposure.

## Physiology and Pre-term Newborn Considerations for Inhaled Nitric Oxide Treatment

### Vascular Mechanism of Action

**Figure 1:**
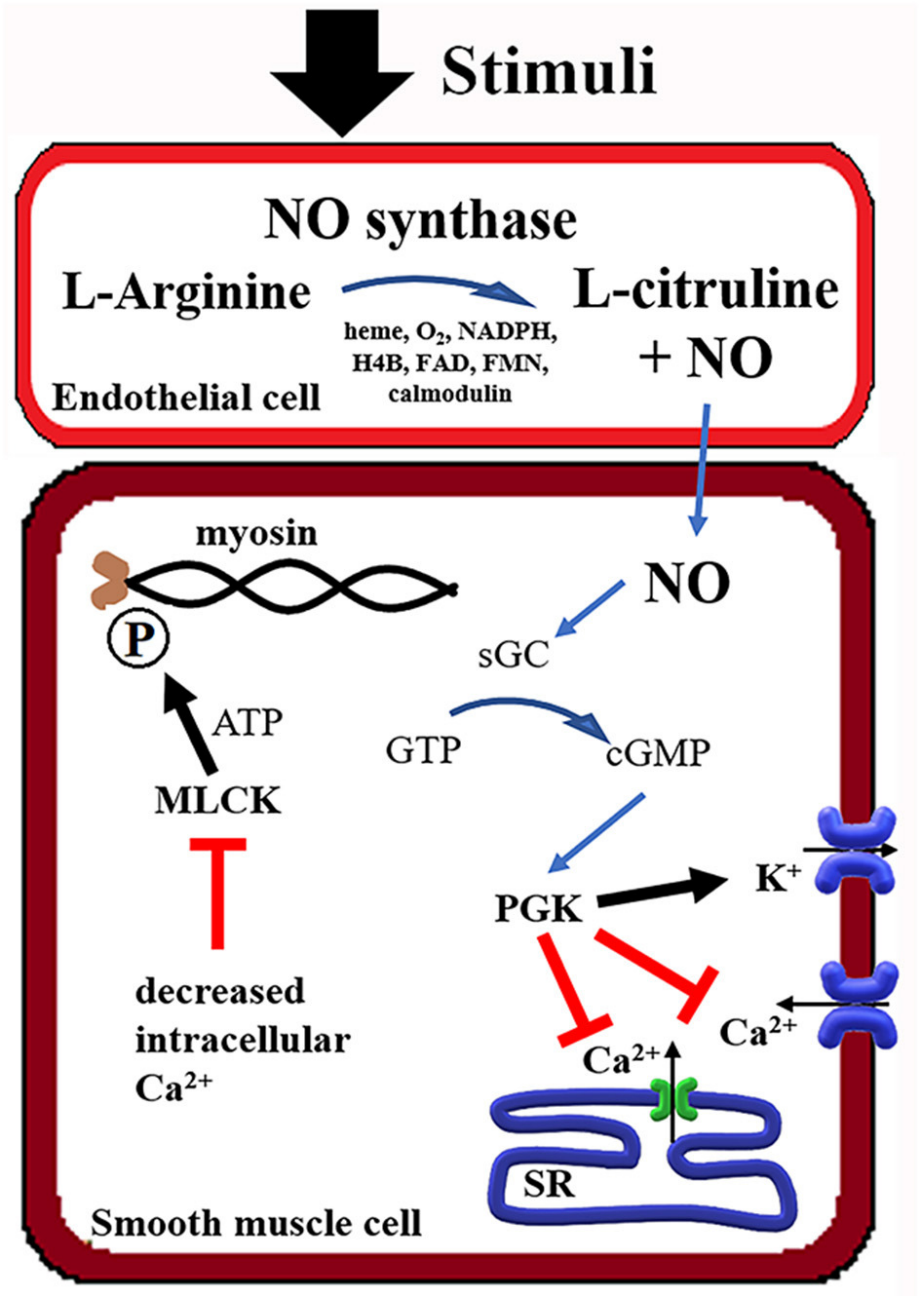
Summary of the Nitric Oxide (NO) mechanism in smooth muscle relaxation. NADPH, Nicotine adenine dinucleotide phosphate; H4B, tetrahydrobiopterin; FAD, flavin adenine dinucleotide; FMN, flavin mononucleotide; GTP, guanosine triphosphate; cGMP, cyclic guanosine monophosphate; sGC, soluble guanylate cyclase; PGK, phosphoglycerate kinase; SR, sarcoplasmic reticulum; MLCK, myosin light-chain kinase; ATP, adenosine triphosphate.

Nitric oxide released by endothelial cells, which covers the entire vascular system, acts on adjacent smooth muscle cells by paracrine signaling, causing vasodilation (20, 21). Nitric oxide is produced by nitric oxide synthase (NOS), which has three different gene isoforms. Neuronal NO synthase (nNOS), cytokine-inducible NOS (iNOS), and endothelial NOS (eNOS), as the name suggests, have a preferential expression, but all participate in vascular tone regulation (22). Different stimuli, including acetylcholine, bradykinin, histamine (calcium-dependent), and shear stress, 17β-estradiol (calcium-independent), induce NOS phosphorylation and production of nitric oxide. NO is generated when L-arginine is converted to L-citrulline, which requires multiple co-factors as heme group, oxygen (O_2_), nicotine adenine dinucleotide phosphate (NADPH), tetrahydrobiopterin (H4B), flavin adenine dinucleotide (FAD), flavin mononucleotide (FMN), and calmodulin (23–27). In the smooth muscle cells, nitric oxide combines with soluble guanylyl cyclase (sGC) and promotes the formation of cyclic guanosine monophosphate (cGMP) (28). Cyclic GMP mobilizes protein kinase G, decreasing intracellular calcium by stimulating calcium reuptake by the sarcoplasmic reticulum (SR) and opening calcium-activated potassium channels (29). The reduction of intracellular calcium decreases phosphorylation of myosin by myosin light chain kinase (MLCK), causing muscle relaxation (30, 31) (Figure 1). The arterial vasodilation effect of iNO in pulmonary circulation maximizes perfusion in well-aerated airspaces, improving oxygenation, and decreasing ventilation-perfusion mismatch (32).

Inhaled nitric oxide supposedly has a half-life of 2–6 s since it is degraded by various mechanisms (33–35). However, it directly affects the lung endothelium by diffusion, reaching the alveolar lining (36). As pre-term newborns have limited peripheral vascular tone control and may present with left ventricular dysfunction, side effects, including systemic hypoperfusion, must be considered during iNO administration (37–40).

### Other Effects of Nitric Oxide

Nitric oxide has multiple effects on cellular function, which can be protective or detrimental in pre-term newborns during major organogenesis (41). These conflicting results demonstrate that iNO should be used judiciously in clinical practice. The most complicating factor is that most of the studies were conducted *in vitro* or in animal models that are not easily extrapolated to pre-term human newborns.

#### Toxicity vs. Protective Effective

Nitric oxide is a free radical (NOx). In mammalian tissues, it can be converted to other toxic compounds, including peroxynitrites (ONOO^−^), nitrogen dioxide (NO_2_), nitrous acid (HNO_2_), and S-nitrosothiols (SNOs). Such compounds cause DNA breakage, lipid peroxidation, protein oxidation, protein nitration, and mitochondrial respiratory enzyme inhibition, leading to apoptosis and necrosis (42–51). One important discussion in pre-term infants is the interaction between nitric oxide and surfactant. In animal experiments with iNO exposure >48 h at doses of > 80 ppm, there was a decrease in surfactant adsorption. In contrast, the surfactant's lipid peroxidation was decreased in isolated surfactant complex exposed to nitric oxide during surface cycling due to reduced conversion of surfactant to small vesicles (52). It is worth noticing, in term newborns, no significant surfactant function change, cytokine profile, or lipid peroxidation has been identified in analyzed airway specimens after iNO treatment. Furthermore, one study detected cell damage markers related to nitric oxide 10 days after the end of iNO treatment, which could be related to endogenous nitric oxide other than iNO (53). Another toxic iNO effect is methemoglobin (metHb) (54). Term and pre-term infants are at higher risk for this condition due to easier fetal hemoglobin oxidation and metHb reductase system immaturity (55, 56). However, metHb above 5% (outside safety range) is rare in newborns of all gestational ages (GA), as long as the iNO dose is below 40 ppm (57–59).

On the other hand, in multiple *in vitro* and animal studies, nitric oxide had protective effects according to environmental factors. Endogenous NO produced in response to reactive oxygen species due to pathogens and pathophysiological conditions served as a free radical scavenger and decreased peroxide-mediated cell damage (51, 60–62). Furthermore, in mature rats and 1-month-old ventilated pigs, iNO decreased inflammatory response by inhibiting the NFκβ pathway (63, 64). In another rat model, iNO reduced leukocyte adhesion and vascular permeability dysfunction in the mesenteric venules (65). Also, in a swine model of cardiopulmonary bypass, iNO decreased interleukin−8 (a chemoattractant) and induced early programmed apoptosis, reducing long-term inflammation (66, 67). The inflammatory regulation of iNO may be beneficial in combating pathogens in the airway. In a rat model of pneumonia, iNO decreased signs of inflammation and improved bacterial clearance (68). In another study in patients with cystic fibrosis and antibiotic resistance microbes, clinical improvement was noticed after iNO exposure (69).

#### Cellular Effects

Nitric oxide can regulate multiple mechanisms and cellular functions, as shown in *in vitro* animal model studies. For example, Cook et al. and Nakaki et al. showed that NO-producing vasodilators decrease DNA synthesis through cAMP and cGMP signaling (70, 71). Furthermore, in rat's smooth muscle cells, induction of endogenous NO played a role in the depression of growth cells (72, 73). In other *in vitro* studies, NO-releasing agents interfered with essential immunologic system pathways by inhibiting cytochrome P450 (74, 75). Even though these studies were not related to NO's inhaled administration, a similar mechanism occurs *in vivo* after iNO exposure in the lung endothelium and smooth muscle.

Another considerable iNO side effect is the modification of platelet function with increased bleeding time in newborns, as shown by George et al. (76). Such an effect can predispose pre-term patients to intracranial hemorrhage, pulmonary edema, and pulmonary hemorrhage. However, a metanalysis of pre-term infants exposed to iNO had no increased complications from bleeding (77).

#### Alveolar and Lung Development

Animal trials of iNO showed a promising effect in improving lung development, which drove clinical trials of the inhaled treatment in pre-term newborns. Following are some of the findings that justified such human trials. Newborn rats exposed to hyperoxia have impaired alveolarization. The use of iNO restored growth factors known to participate in developing the airway, like fibroblast growth factor and vascular endothelial growth factor (78, 79). Curiously, Lopez et al. showed rats treated with iNO had similar mortality, lower weight gain, and capillary alveolar density than non-treated animals (78). On the other hand, Lin et al. showed enhanced lung growth and alveolarization in the recovery period if animals were treated with iNO at 10 ppm (79).

In animal models of pre-term ventilated lambs, Kinsella et al. exposed their animals to iNO for a short period of time after birth (3–4 h). Treated animals had a decrease in neutrophil migration, no significant changes in vascular permeability, and improved gas exchange (80). These findings were expanded by Bland et al. with a model of chronic lung disease. Lambs were ventilated and treated for 3 weeks. Animals in the iNO group had improved radial alveolar count and capillary surface density (81).

McCurnin et al. applied a similar strategy to a baboon model of neonatal chronic lung disease. Improvement was noticed in pulmonary-to-systemic blood flow ratio (Qp/Qs), ventilation index, lung compliance, and expiratory resistance, mostly in the first 8–10 days after iNO exposure. Yet, after the day of life 10, there was no statistical significance, indicating the initial benefit only for early and short use of iNO, but not chronic administration. However, lung weight and DNA content were improved after the study if animals were treated with iNO for a total of 14 days (82).

#### Cardiovascular Effects

**Figure 2:**
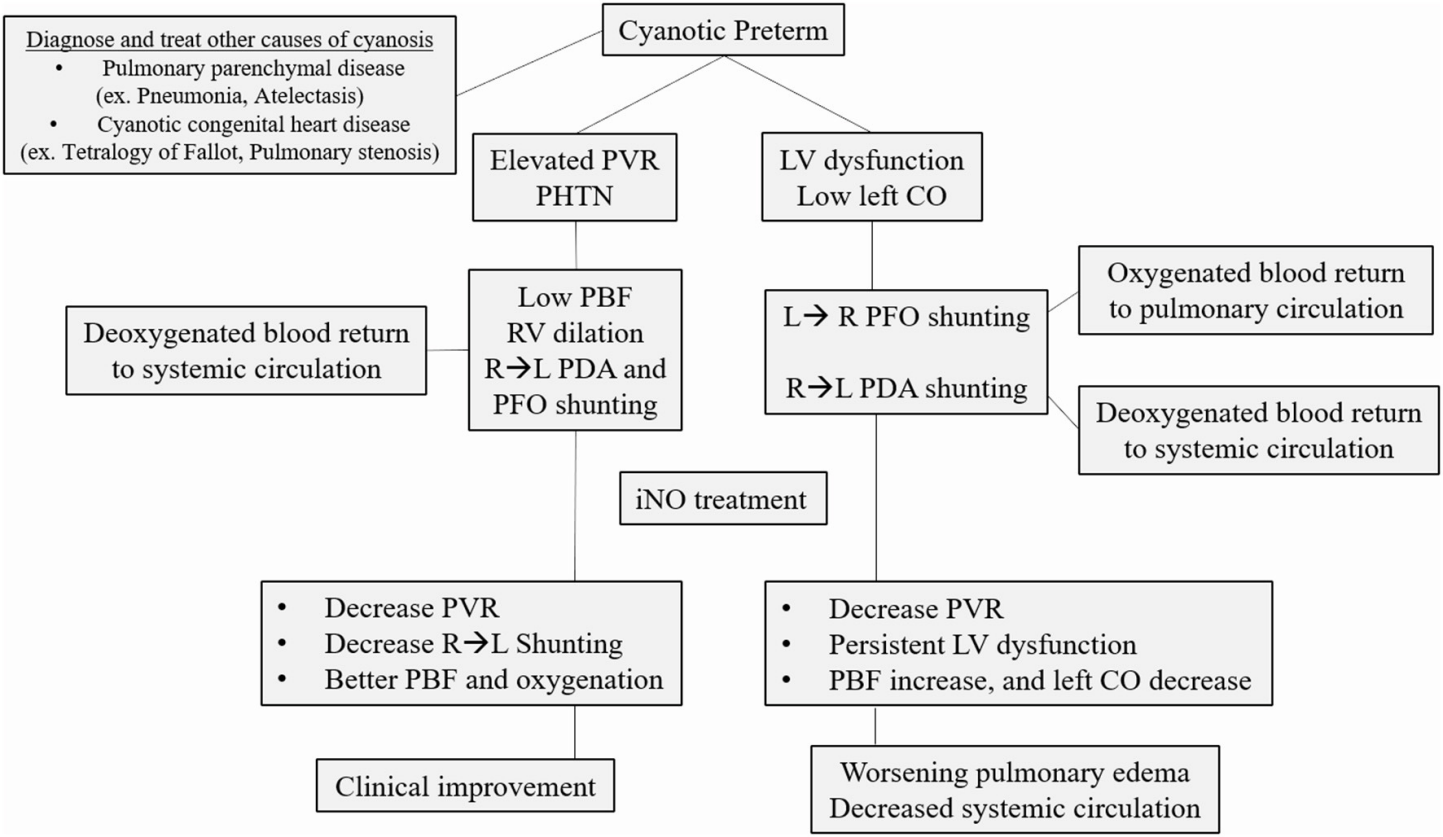
Common causes of cyanosis in the pre-term infant. After ruling out pulmonary parenchymal disease and cyanotic congenital heart disease, the clinician should determine the primary cause of hypoxic respiratory failure. If elevated pulmonary vascular resistance (PVR) with a right to left shunt (RL) is the primary determinant of cyanosis, inhaled nitric oxide (iNO) is the recommended treatment. If there is significant left ventricle (LV) dysfunction, not related to RV dilation from PHTN, iNO can decrease left cardiac output (CO) even further, worsening the clinical status. PBF, Pulmonary blood flow; PHTN, pulmonary hypertension; PFO, patent foramen ovale; PDA, patent ductus arteriosus.

In adult studies, when used in pulmonary hypertension associated with right heart failure, inhaled nitric oxide has been shown to lower pulmonary vascular resistance (PVR) and improve left ventricle (LV) output (83). However, when used in patients with LV dysfunction, the resultant increase in pulmonary venous blood flow further compromised LV function and decreased cardiac output. One exception is LV dysfunction related to RV dilation due to PHTN and major bulging to the interventricular wall compressing the LV cavity (84). For this reason, neonatologists have to understand the hemodynamics and the cause of the hypoxic failure before using iNO. Patients with high PVR causing right-to-left shunt at the PDA level will benefit from pulmonary vasodilator as iNO. If the right to left shunt is related to LV dysfunction or low systemic vascular resistance (SVR), inotropic drugs and volume expansion can reverse this shunt by increasing arterial blood pressure, and iNO could be detrimental if used as a primary treatment agent (Figure 2) (85–87). Another consideration, PHTN rebound after iNO discontinuation, is not commonly reported but is a potential complication before full endothelium recovery (88–90).

##### Summary

The nitric oxide role of vascular relaxation is well-determined in the developed lung. Animal and human data still demonstrate the controversial role of iNO in inflammation and toxicity. Furthermore, the clinician should identify the cause of the hypoxic failure in the pre-term infant since iNO can be detrimental if used in patients with primary LV dysfunction.

### Physiologic Considerations of Pulmonary Hypertension in Pre-term Newborns

At any GA, the fetus PVR is elevated compared with systemic vascular resistance (SVR) since the lungs have constricted vessels and are fluid-filled *in utero*. After birth, PVR reduction occurs due to air entry, establishing functional residual capacity, higher PaO_2_, decreased lung fluid, and multiple chemical pathways, including intrinsic nitric oxide (91). The reduction in PVR/SVR ratio diminishes right-left shunting and increases blood flow to the lungs, resulting in improved gas exchange. In situations where this transition is impaired, PHTN may develop, for which iNO is the current standard treatment in late pre-term and term infants (92, 93).

Multiple factors are involved in PVR regulation after birth in pre-term newborns. Nevertheless, the anatomical and physiological lung structure of the pre-term lung may influence iNO response (94–97). Primarily, the endothelium of lung vasculature is underdeveloped, and the lack of intra-acinar arterial muscular layer is the most significant barrier for proper iNO response (98, 99). For example, in fetal lamb studies, increased PVR and response to oxygen are likely related to better vascular smooth muscle development with the advancement of GA (100, 101).

#### Summary

Inhaled NO can facilitate a decrease in PVR by smooth muscle relaxation. However, the pre-term lung vasculature is underdeveloped to respond to the vasodilatory effects of iNO appropriately.

## Major Controversies Of iNO Use in Pre-term Newborns

### Current Recommendations vs. Utilization of iNO in Pre-term Newborns

The existing literature on iNO in term and late-pre-term newborns in the presence of PHTN has raised a question about the possible benefits of the same drug in newborns ≤ 34 weeks GA. Early case reports proposed the benefits of iNO in pre-term infants with HRF (102–105). As discussed above, some animal models of lung injury have shown positive results, with enhanced lung growth and development, when exposed to iNO (78–81). These findings suggest that iNO could be used as a preventive agent for bronchopulmonary dysplasia (BPD). On the other hand, no pre-clinical trial has investigated the ideal GA to offer iNO since pre-term lungs may lack the structures necessary to respond to the treatment properly (101).

Prematurity-related complications are not decreasing because of increased survival of extremely pre-term infants (106), including infants with BPD (107, 108). According to the Institute of Medicine, prematurity's overall financial burden reached $26 billion in 2005. To combat this increased burden, many investigators focused on overcoming these complications in pre-term neonates by utilizing iNO to prevent BPD, as well as a treatment for acute HRF.

#### Current Recommendations

The first randomized placebo-controlled iNO trials were published in 1999. The results demonstrated the efficacy of iNO in improving oxygenation in HRF, but it failed to improve survival or BPD in pre-term newborns (109, 110). Other randomized trials have been published with similar or contradictory results, leading to the first Cochrane review in 2010. This systematic review was divided into three categories based on initial iNO exposure and the following criteria; (1) initial 3 days of life based on hypoxia criteria, (2) initial 3 days due to pulmonary disease, and (3) later recruitment according to an elevated risk of BPD. No statistically significant positive results were found in any of these groups for decreases in BPD, infant mortality, or adverse events, such as an increase in intraventricular hemorrhage (IVH) or neurodevelopmental impairment (111). Besides, two other independent comprehensive data reviews, JHU EPC and MAPPiNO, found no significant benefits for the pre-term newborns exposed to iNO (112, 113).

It should be noted that these studies varied in iNO dosing, age at first dose, and treatment duration. Despite these irregularities, an NIH Consensus and the Committee on Fetus and Newborn published a policy statement that the current data do not support the routine use of iNO in pre-term infants (114, 115). The consensus made it clear that subpopulation analysis, including demographics (gestational age, ethnic groups) and medical factors (pulmonary hypertension and lung hypoplasia), were not sufficiently evaluated. Therefore, targeted clinical trials are needed with predefined subgroups (116). The latest Cochrane review concluded that no significant benefits are gained when pre-term infants are exposed to iNO. As with the earlier studies, these studies did not evaluate the use of iNO for common medical conditions, such as PHTN, in pre-term newborns (117).

#### Increasing Use of iNO in Pre-term Newborns

Inconsistent with published recommendations, 2016 data from California showed that 2.61% of newborns ≤ 34 weeks GA had been treated with iNO. The highest percentage of utilization was in newborns with gestational ages from 22 to 24 6/7 weeks GA (10.6% in regional centers). Furthermore, newborns < 27 weeks GA had the highest increase in iNO exposure since 2007 (118).

According to Manja et al. a national survey showed that most neonatologists continue to prescribe iNO to pre-term newborns based on the imperative that physicians should “do something/do everything” to treat HRF on maximal ventilatory support (119). Taken together, these data make clear that clinicians remain uncertain about the possible benefits of iNO in the vulnerable population of extremely pre-term infants, mainly due to a lack of studies on its efficacy in treating specific medical conditions in this population. This underscores the importance of specifically targeted clinical trials that are needed to resolve this uncertainty.

##### Summary

Despite the lack of evidence for using iNO in pre-term newborns, the use of iNO continues to increase.

### Pulmonary Hypertension Diagnosis in Premature Newborns

The benefits of iNO in pulmonary hypertension in term infants are very well-described. However, diagnosis and characterization of pulmonary hypertension in premature neonates are incredibly challenging due to the presence of intra- (patent foramen ovale-PFO) or extra-cardiac shunts (patent ductus arteriosus–PDA) and continued change in hemodynamics according to gestational age. Multiple echocardiography parameters have been beneficial as modified shunt blood flow allows accurate hemodynamic assessment and can be used to follow response to the prescribed therapy.

**Figure 3:**
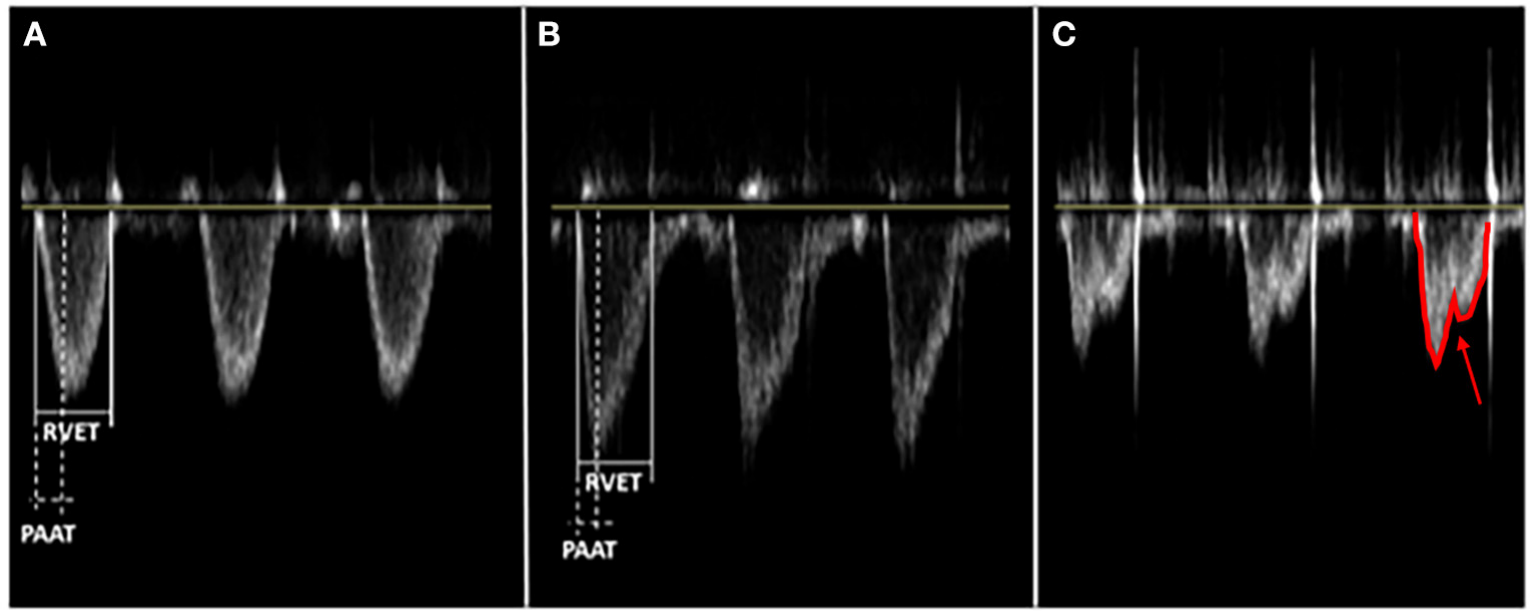
Different main pulmonary artery (MPA) flow velocity profile patterns depend on the pulmonary vascular resistance (PVR). PVR is an index ratio of right ventricular ejection time (RVET) and pulmonary artery acceleration time (PAAT), both obtained from the right outflow tract's pulse-wave doppler. PAAT, as shown, is the interval from the onset of ejection through the pulmonic valve to the peak flow velocity. These are three distinct patterns of flow velocity envelope: **(A)** Parabolic or normal PVR - Isosceles triangle, RVET:PAAT <4; **(B)** Right-angle triangle or increased PVR - RVET:PAAT >4; **(C)** Notched pulmonary artery doppler or severe increase in PVR - two distinct peaks (arrow) from midsystolic flow deceleration.

PHTN is consistent with a right-left communication or a bidirectional PDA shunt >60% right-to-left (120). When shunts are not present or conclusive, other indirect measures, such as tricuspid regurgitation jet, leftward/flattened intraventricular septum, right ventricular dysfunction or dilation, and low left ventricular output, are used to indicate the presence of PHTN. Unfortunately, there is no normative data for post-conception age due to the multitude of factors implicated in shunt direction as systemic blood pressure, PFO size, right and left ventricular function, and pathology causing elevated PVR (85). In 2016, Levy et al. used echocardiogram-derived pulmonary artery acceleration time (PAAT) with 95% feasibility, 97% sensitivity, and 95% specificity when compared with right heart catheterization in diagnosing children (121). In 2019, Patel et al. demonstrated that PAAT could reliably be used in pre-term newborns (122). Due to the recent validation of PAAT, no studies presented in this review used PAAT as criteria for diagnosing PHTN (Figure 3).

Considering that the echocardiography diagnosis of PHTN is challenging in pre-term infants, adding clinical and echocardiography parameters may maximize the accuracy of diagnosing PHTN (85). The classical findings of a differential oxygen saturation >10% between pre-and post-ductal with pre-ductal being greater is often used to diagnose the presence of right-to-left shunts (85). The absence of differential saturation does not exclude the presence of a right-to-left shunt if the shunting is predominantly at the atrial level.

In recent years, the combination of clinical findings and echocardiogram results triggered the development of targeted neonatal echocardiography (TnECHO) or functional echocardiogram by neonatologists. This patient's overall evaluation provided novel physiologic insights and permitted individualized care of these neonates. For example, Ahmed et al. developed a scoring system using echocardiogram findings associated with clinical findings to better diagnose PHTN in pre-term newborns in a cohort study, specifically using the relationship between right ventricular and systolic pressure (123). Such a scoring system requires bedside evaluation of the neonatologist from the TnEcho team. The real-time assessment of the hemodynamic changes, early pathological situations assessment, and response to treatment interventions improves the outcomes of pre-term neonates (124–127). Still, it is essential to mention the importance of the pediatric cardiologist's echocardiogram or review of the echocardiogram done by TnEcho to rule out anatomical congenital heart disease.

### Summary

Diagnosis of PHTN in pre-term newborns clinically or by echocardiogram continues to be controversial, but with the development of TnEcho better outcomes could be expected.

## A Personalized Evaluation and Treatment Of PHTN In Pre-term Newborns

In the era of personalized and precision medicine, prevention and treatment strategies are increasingly sensitive to individual differences (128). One recent application of specialized medicine in the pre-term newborn population is the Askie et al. meta-analysis showing that African-American pre-term newborns respond better to iNO therapy than do infants of other racial groups (129). This finding suggests that large studies with average data across a diverse population, regardless of their unique medical status, can mask the specificity of the benefits in subsets of patients (130). Therefore, we evaluated non-randomized controlled trials (RCT) studies showing the benefits of providing iNO to pre-term newborns. Though the quality of evidence in these non-RCT studies is limited, continued evaluation of available retrospective or prospective data may give new insight into the apparent conflicts between studies and in differing clinical practices.

Following the rationale of precision medicine, we identified and then narrowed the range of clinical indications for which iNO could be recommended to decrease side effects while reducing costs. The rationale that iNO inhalation might benefit a subset of pre-term newborns was based on existing case reports and series. Initially, the investigated pre-term infants presented with PHTN and HRF associated with pre-term premature rupture of membranes (PPROM), as this was proposed to cause PHTN in term and pre-term newborns, possibly by chest compression or decreased distal airway pressure (103, 131–134). However, the observation that iNO and effective ventilation management dramatically improve oxygenation in newborns with a short period of PPROM is not always consistent with the theory of severe lung hypoplasia. In fact, retrospective studies found a correlation of PPROM >120 h as a poor predictor for iNO treatment failure (135, 136). Aikio et al. showed that PPROM patients decreased nitrites and nitrates, which improved after iNO administration. The vasodilation that proposedly improved oxygenation does not explain the increase in lung compliance and ventilation (137). Likely the same relaxation of smooth vascular cells could be applied to acinar smooth muscle cells but has not been evaluated in term and pre-term infants (138). Pre-term infants can still present with similar pathophysiology as term babies due to slow or complicated pulmonary transition, which can benefit from iNO (139).

The limiting factor to this research is that this patient subset (pre-term newborn with acute PHTN) is frequently underdiagnosed if early echocardiogram assessment is not pursued. With the advancement of TnECHO techniques, placebo-controlled clinical will be possible, as shown in recent PDA treatment trials (140). One example, Cheng et al. utilized TnECHO in newborns <34 weeks GA to determine early PHTN diagnosis (141). Their findings were consistent with other studies showing that early iNO initiation in pre-term newborns more than 1,000 g produce better outcomes than does in smaller newborns with late-onset HRF attributable to sepsis

Another critical factor is the safety of iNO in pre-term infants. Both Carey et al. and Ellsworth et al. indicated that pre-term newborns receiving iNO within 7 days of birth had no increase in mortality (142, 143). Furthermore, most of the studies presented here reinforced the safety of iNO, even when no benefit was found.

The findings of these non-randomized studies, however, remain equivocal. To provide maximal clarity, they will be grouped and reviewed by the following characteristics: GA and BW, chronological age, inclusion criteria, dose of iNO, clinical features that predict better iNO response, and outcomes. This will allow readers to apply these findings to clinical practice appropriately. Table 1 summarizes the key elements of each reference.

**Table 1:** Characteristics of pre-term infants that responded to iNO treatment.

**References**	**Type of study**	**GA and/or weight**	***N***	**Inclusion criteria**	**PHTN on echo**	**Intervention**	**Significant findings**	**Major weakness**
Uga et al. (144)	Retrospective comparative study	<1,500 g	18	PPROM >5 days; HRF; PEEP>8	7 out of 7	iNO 30 ppm, up to 40 ppm; < 24 h of life	iNO increased PaO2 and survival at 28 days	Small N; no echocardiogram in controls.
Kumar et al. (145)	Retrospective, case-control analysis	<37 wks	61	Echocardiogram evidence of PHTN up to 4 weeks of life	61	iNO 5 ppm, up to 15 ppm	Newborns >1,000 g were more likely to respond to iNO; Non-responders have higher mortality	No echocardiogram in the control group.
Chock et al. (146)	Retrospective *post hoc* subset analysis from PiNO trial & larger preemie pilot	<34 wks, <1,500 g (PiNO) & <34 wks >1,500 g (large preemie)	12	Pulmonary hypoplasia; PPROM or oligohydramnios. iNO vs. O^2^ placebo	4 out of 5	iNO 5 ppm, up to 10 ppm	iNO group: increased PaO2; decreased ventilation and oxygen days	Small N; echocardiogram not done routinely.
Chandrasekharan et al. (136)	Retrospective comparative Study	<34 wk	93	iNO use in first 28 days of life	Not clear	iNO 20 ppm; < 28 days of life	iNO responders, responded early with better survival. PPROM and antenatal steroids predicts survivors	Echocardiogram not done routinely.
Baczynski et al. (135)	Retrospective cohort study	<35 weeks	89	iNO for early PHTN at <3 days of life	Echocardiogram done, but not detailed	iNO 20 ppm; <3 days of age after acute PHTN was diagnosed by echocardiogram or clinical diagnosis	Responders: females, PPROM, received surfactant, 1st DOL, PaO2 improvement at 1 h; responders had higher survival and lower disability.	No control group, echocardiogram not done routinely.
Dani et al. (147)	Retrospective cohort Study	<30 wks, <1,250 g	42	Severe RDS despite surfactant	28 out of 42	iNO 20 ppm, up to 40 ppm	PHTN group had faster and better improvement after iNO; responders had a higher birth weight (>750 g), FiO2 ≥ 0.65, PPROM did not have a better response	Small N to evaluate responder vs. non-responders with or without PHTN.
Rallis et al. (148)	Retrospective cohort Study	<34 wks	55	HFR with evidence of PHTN	52 out of 52	iNO 5 ppm, up to 20 ppm. PHTN by echocardiogram or clinical diagnosis	PHTN and oligohydramnios had better response; early PHTN (<72 h) had higher survival. PPROM alone did not have a better response to iNO	No control group
Kettle et al. (149)	Retrospective observational study	< 34 wks	72	Pulmonary hypoplasia treated with iNO	30 out of 44	iNO 20 ppm	Non-responders had higher mortality. PHTN by echo did not predict response to iNO	Voluntary data submission to a registry. No control group. No clear definition of pulmonary hypoplasia.
Rhine et al. (150)	Retrospective registry analysis	< 34 wks, mean of 27.1 wks GA	431	Received iNO ≤ 7 days of life	Echocardiogram done, but not detailed	iNO 20 ppm	60% improvement, mostly in the 1st h. 99.5% presented with HRF and PHTN.	No control group. Echocardiographic findings not described.
Ahmed et al. (123)	Retrospective Cohort study	<36 weeks	213	FiO2 ≥ 0.6, OI ≥ 10, Echocardiogram within 24 h of iNO initiation	53 out of 73	Targeted neonatal echocardiogram (TnECHO) followed by iNO and vasopressors/inotropes	Presence of PHTN on echo and treatment before 72 h of life had better response. IVH III/IV higher in the iNO group.	TnECHO not done in all patients. Head US not standardized.

*N, number*.

### Gestational Age and Birth Weight

This discussion focuses on pre-term newborns <34 weeks GA and <2,500 g. However, some of the retrospective studies also addressed BW limits that involved late-pre-term newborns. In all studies, the most common finding was that newborns with birth weight above 750 g were more likely to benefit from iNO administration. *Post-hoc* analysis of two large RCTs, the PINO trial and the Large Preemie Pilot trial, found that newborns with BW >1,000 g had lower mortality than newborns with lower BW (146). Other retrospective studies suggested that newborns>1,000 g were more likely to benefit from iNO (136, 145). Conversely, three studies reported that newborns as small as 750 grams could still benefit from iNO, likely because of receiving surfactant before iNO initiation (123, 135, 147). Since most studies focused on birthweight, few studies mentioned GA as a limiting factor. The three studies' threshold was around 27–29 weeks, which could correlate with lower BW, as discussed above (136, 145, 150).

#### Summary

Newborns with a birth weight of ≥750 g and older than 27 0/7 weeks GA may benefit from iNO.

### Time and Duration of iNO Administration

The goal of all non-RCTs presented in Table 1 was to evaluate the effect of iNO on acute PHTN and HRF. Consequently, newborns receiving iNO for BPD prevention and BPD with PHTN were excluded. The patients analyzed were under 4 weeks of age. Pre-term newborns benefitting most from iNO administration were those treated between the first hour of life and 3 days of life (123, 135, 144, 147, 148). Treatment duration also correlated with hypoxia improvement after treatment initiation. The included studies varied in the criteria used to indicate a clinical response to iNO administration; however, a decrease in FiO_2_ by 0.15–0.30 or an increase in PaO_2_ by at least 20 mmHg were the most common outcome measures. OI was not reported in all studies. Kettle et al. indicated a median improvement in FiO_2_, PaO_2_, and OI, even when non-responders were included (149). Most studies reported clinical response latencies between 30 min and 6 h of treatment onset (135, 145, 147–150). Due to its short half-life and rapid clinical effect, FiO_2_ and PaO_2_ should be assessed no later than 6 h after iNO treatment initiation. iNO discontinuation and dose adjustments should be considered to decrease potential side effects and eliminate unnecessary costs in newborns with inadequate iNO treatment response.

#### Summary

iNO should be started early in the disease course, preferentially before 72 h of life. Evaluation of treatment response should be done within the first 6 h of treatment.

### Inhaled Nitric Oxide Dosing

The most common iNO dosing in the studies analyzed was between 10 and 20 ppm due to the risk of methemoglobinemia with higher dosing (57, 58). iNO dosing was extensively evaluated in multiple RCTs; dosing outside this range should be closely monitored (117). Only one study, Uga et al. used a higher dose range, between 30 and 40 ppm. However, this group did not find an increased incidence of IVH, BPD, or metHb above 2% in the treatment group (144). Three of the studies analyzed iNO dosing as low as 5 ppm. These protocols adjusted doses up to 20 ppm. Except by Rallis et al. (protocol increased up to 20 ppm in all patients), it is unclear how many patients had iNO increased to the maximum dose or if any clinical benefits resulted from iNO adjustment (145, 146, 148).

#### Summary

iNO dosing should begin between 5 and 10 ppm in pre-term infants. There is no evidence of clinical benefits with dosing above 20 ppm, and the risk for side effects increases at higher doses.

### Clinical Characteristics Predicting Improvement After iNO Treatment

Identifying the best iNO response predictor in pre-term newborns varies among studies. Differential inclusion criteria and research methodologies (cohort vs. case-control) prevent the identification of reliable recommendations. For example, Uga et al. and Chock et al. used PPROM as a typical inclusion criterion. While most patients received an ECHO to document PHTN, ECHOs were not always done on controls. In both studies, iNO was shown to improve PaO_2_ with increased survival and decreased ventilator support days (144, 146). Chandrasekharan et al. and Baczynski et al. followed pre-term newborns receiving iNO from birth and reported outcomes. Both studies identified PRROM >18 h as a significant predictor for improvement after iNO. Interestingly, patients with PPROM exceeding 120 h had lower iNO treatment response (135, 136). Kumar et al. proposed that patients exposed to PPROM with oligohydramnios and low Apgar scores were more likely to develop PHTN. Newborns with PHTN responded better to iNO treatment, with higher survival rates than non-responders in all conditions except sepsis-induced PHTN (145). Conversely, studies focusing on HRF and PHTN as inclusion criteria did not consistently identify PPROM as a common characteristic of iNO responders. For example, Dani et al. and Rallis et al. evaluated patients with HRF or severe RDS despite surfactant administration. Patients diagnosed with PHTN had a better iNO response than non-PHTN patients. However, PPROM did not predict iNO response. Alternatively, oligohydramnios was a positive predictor of iNO treatment (147, 148). It should be noted that not all patients with PPROM develop pulmonary hypoplasia unless they have prolonged oligohydramnios.

One obvious but often overlooked finding is that pre-term newborns with PHTN were more likely to respond to iNO treatment than were newborns without ECHO confirmed PHTN (123, 147). Also, patients exposed to prenatal steroids and receiving surfactant postnatally had better iNO responses, possibly related to improved ventilation from surfactant, ultimately maximizing ventilation-perfusion (V/Q) ratio (135, 136). Clinicians dealing with pre-term newborns progressing toward HRF should also know that FiO_2_ of 1.0 is not required before considering iNO. Dani et al. showed significant improvement in pre-term newborns receiving FiO_2_ between 0.6 and 1.0 with a baseline of 0.77 (147). This provides evidence for starting iNO therapy before PHTN advances to an irreversible status.

#### Summary

Pre-term newborns with PPROM, oligohydramnios, and echocardiography or clinical PHTN diagnosis are more likely to benefit from iNO if they progress to HRF. The surfactant should be given before iNO if clinically indicated. iNO should be started before FiO_2_ reaches 1.0.

### Positive iNO Response Predicts Better Outcomes

The current literature shows a positive iNO response (PiR) from 43 to 78% in pre-term newborns with HRF and even better response rates for newborns with PHTN diagnosis who receive early iNO treatment (123, 135, 136, 147, 148, 150). However, not all studies included long-term outcomes other than clinical improvements, such as FiO_2_ decrease or PaO_2_ increase. Studies analyzing long-term disabilities and survival showed that PiR experienced benefits in addition to oxygenation improvement.

Survival rates for PiR exceeded those of iNO non-responders (NiR) in four different studies. Survival rates were 74 vs. 33% (148), 88 vs. 70% (136), 80 vs. 41% (149), and 66 vs. 29%, respectively, for PiR and NiR (135). The latter study further compared mortality by comparing early (<7 days) and late (>7 days) iNO exposure, finding significantly higher survival rates for those receiving iNO in the 1st week. Furthermore, PiR had lower IVH grades and PVL (2.8 vs. 28.5%) and higher disability free-survival at 18 months (51 vs. 15%) (135, 136). Other comorbidities, such as cerebral palsy and chronic lung disease, were similar in PiR and NiR (135, 149).

#### Summary

iNO responders are more likely to survive than were non-responders. Responders may experience lower neurodevelopmental disability rates but with similar chronic lung disease rates.

## Novel iNO Use in Premature Newborn Resuscitation

This review found that many controversies remain in pre-term newborn care, including delivery room resuscitation. Presently, optimal FiO_2_ in premature newborns during resuscitation is unknown (151). Current resuscitation guidelines recommend maintaining target oxygen saturation for both term and pre-term infants (152, 153). Despite this, pre-term infants may be exposed to high oxygen concentration during resuscitation to maintain optimal oxygen saturation targets (154). The morbidities associated with increased oxygen concentration exposure in premature infants are well-known (155). The primary emphasis of current NRP guidelines is on ventilation to facilitate pulmonary vasodilation. However, there is no alternative to such measures for non-responders. Hence, iNO may be an adjunct therapy to decrease PVR and reduce excessive oxygen exposure. In the neonatal lamb model of asphyxia and studies of premature newborns, lower FiO_2_ use during resuscitation after birth decreased oxidative stress (156, 157). Therefore, the administration of iNO may facilitate less oxygen exposure to premature newborns during neonatal resuscitation by improving V/Q mismatch. This novel hypothesis was studied in a randomized controlled pilot trial in premature newborn infants between 25 0/7 and 31 6/7 weeks GA. Premature infants were randomized to oxygen + nitrogen as a placebo or oxygen + iNO starting at 20 ppm and titrated down during the first 17 min of resuscitation. The results showed a trend toward lower oxygen exposure in the oxygen + iNO group, with significantly less cumulative oxygen exposure than the oxygen-only group. Besides, the placebo group had significantly higher exposure to hyperoxia (FiO_2_ > 60%) than did the treatment group. There were no differences in the secondary outcomes measured, including IVH. This first pilot human trial suggested that the administration of iNO during resuscitation is feasible and decreased exposure to supplemental oxygen in premature infants (158). It should be noted that an acute reduction in PVR during the transition with iNO may increase the pulmonary venous return and increase organ perfusion, particularly to the brain, increasing the risk of IVH. Therefore, caution should be exercised when iNO is administered during early adaptation. A larger randomized trial is needed to validate this novel approach further.

## Conclusion

Non-randomized controlled trials suggest that inhaled nitric oxide can be a treatment option for pre-term newborns with hypoxic-respiratory failure associated with pulmonary hypertension. To achieve better outcomes, we advocate for early assessment with echocardiogram or TnECHO for the certainty of diagnosis and prompt treatment with iNO. However, the debate on the risks and benefits of iNO in pre-term newborns is far from over. Investigations of pathophysiology, pharmacology, and pharmacogenetics are needed to validate such treatment in pre-term newborns with pulmonary hypertension.

## Author Contributions

FV, MM, and KS contributed to the concept, design, literature review, and writing of the article. All authors contributed to the article and approved the submitted version.

## Conflict of Interest

KS has received research grants from Mallinckrodt and Pfizer Pharmaceuticals. The remaining authors declare that the research was conducted in the absence of any commercial or financial relationships that could be construed as a potential conflict of interest.
